# Development of a Matrix for Seismic Isolators Using Recycled Rubber from Vehicle Tires

**DOI:** 10.3390/polym16212977

**Published:** 2024-10-24

**Authors:** Alex Oswaldo Meza-Muñoz, Faider Sebastian Rivas-Ordoñez, Ingrid Elizabeth Madera-Sierra, Manuel Alejandro Rojas-Manzano, Edwin Dielmig Patino-Reyes, Manuel Iván Salmerón-Becerra, Shirley J. Dyke

**Affiliations:** 1 Departamento de Ingeniería Civil e Industrial, Pontificia Universidad Javeriana Cali, Calle 18 No. 118-200, Santiago de Cali 760031, Colombia; 1004561533@javerianacali.edu.co (F.S.R.-O.); alejandro.rojas@javerianacali.edu.co (M.A.R.-M.); 2 Facultad de Artes Integradas, Departamento de Tecnología de la Construcción, Universidad del Valle, Calle 13 No. 100-00, Santiago de Cali 760042, Colombia; ingrid.madera@correounivalle.edu.co; 3 Lyles School of Civil Engineering, Purdue University, 585 Purdue Mall, West Lafayette, IN 47907-2088, USA; patino7@purdue.edu (E.D.P.-R.); salmeron@purdue.edu (M.I.S.-B.); sdyke@purdue.edu (S.J.D.); 4 School of Mechanical Engineering, Purdue University, 585 Purdue Mall, West Lafayette, IN 47907-2088, USA

**Keywords:** elastomeric seismic isolator, recycled rubber, matrix, tires

## Abstract

Over recent decades, numerous strong earthquakes have caused widespread devastation, including citywide destruction, significant loss of life, and severe structural damage. Seismic base isolation is a well-established method for mitigating earthquake-induced risks in buildings; however, its high cost often limits its implementation in developing countries. Simultaneously, the global rise in vehicle numbers has led to the accumulation of discarded tires, intensifying environmental challenges. In response to these issues, this study investigates the development of a seismic isolator matrix using recycled rubber from vehicle tires, proposed as a sustainable and cost-effective alternative. Ten recycled rubber matrices were experimentally evaluated for their physical and mechanical properties. The matrix with optimal granulometry and binder content, demonstrating superior performance, was identified. This optimized matrix underwent further validation through compression and cyclic shear tests on reduced-scale prototypes of fiber-reinforced isolators, which included five prototype designs, two of which featured flexible reinforcement. The best-performing prototype comprised a recycled rubber matrix with 15% binder and glass fiber, exhibiting vertical stiffness and damping characteristics superior to those of natural rubber. Specifically, this prototype achieved a damping ratio of up to 22%, surpassing the 10% minimum required for seismic isolation, along with a vertical stiffness of 45 kN/mm, critical for withstanding the vertical loads transferred by buildings. These findings suggest that the recycled tire rubber matrix, when combined with glass fiber, is a viable material for the production of seismic isolators. This combination utilizes discarded materials, contributing to environmental sustainability.

## 1. Introduction

Seismic events occur regularly as a result of the release of energy from the Earth’s crust, varying from minor tremors to those powerful enough to devastate cities, damage structures, and cause significant human and material losses. Consequently, civil engineers bear the crucial responsibility of designing and constructing buildings in accordance with established design standards. They apply advanced engineering principles to safeguard human lives and minimize structural damage. In this context, energy dissipation and seismic isolation systems have been adopted as passive mechanisms for mitigating seismic responses [1].

Base seismic isolation systems are among the most effective approaches to date [1,2]. These isolators consist of an elastomeric matrix and reinforcement, capable of significantly reducing the transfer of seismic movements from the ground to the structure [1,2]. During a seismic event, the elastomeric matrix absorbs and dissipates energy by deforming, while the reinforcement provides additional strength and structural stability against vertical loads [3]. Steel-reinforced elastomeric isolators (SREI) are seldom used in developing countries due to their size, weight, and high cost associated with complex manufacturing processes [3,4].

To address these issues, more economical isolators could be utilized by simplifying the installation process, reducing energy consumption in manufacturing, and using common or recycled raw materials [4]. Consequently, fiber-reinforced elastomeric isolators have been developed as cost-effective alternatives. These isolators can be either connected to a structure (FREI) or Unbonded (U-FREI). This alternative significantly reduces weight and cost, as the use of fiber allows for a simpler manufacturing process and requires less labor [5,6,7].

Madera et al. developed low-cost High Damping Rubber U-FREIs (HDR) for low-rise residential buildings using a natural rubber matrix, bi-directional polyester fibers as reinforcement, and local labor, this demonstrated that it is a more economical alternative to traditional seismic isolation methods [5,6]. However, U-FREIs exhibit lateral overturning deformations under horizontal loads, where the top and bottom surfaces of the isolator partially detach from the contact support, forming a curved profile [8]. Ghorbi and Toopchi-Nezhad developed hollow circular (HC) isolators to provide greater lateral flexibility [8]. These isolators were made with a natural rubber matrix, reinforced with bi-directional carbon fiber, and featured a central opening representing 19% of their gross area. HC isolators demonstrated 18% lower vertical stiffness, 20–50% higher horizontal flexibility, and significantly higher energy dissipation capacity (in terms of effective damping) compared to U-FREIs [8]. Additionally, a significant issue related to isolators using natural rubber is that their manufacturing process involves vulcanization at temperatures above 140 °C for at least 6 h. This results in high energy consumption and considerable production time. Furthermore, the unregulated production of natural elastomers can have serious environmental impacts, such as the conversion of natural forests into rubber plantations, negatively affecting biodiversity and fragmenting habitats [9]. These challenges raise important questions regarding the efficiency and sustainability in the manufacture of these isolators.

Although conventional seismic isolators typically use natural rubber in their matrix, there is a growing interest in seeking more sustainable alternatives. Evaluating the possibility of replacing natural rubber with recycled rubber in isolator production is important, particularly when considering the issue of end-of-life vehicle tires. This problem is exacerbated by the exponential growth of the global vehicle fleet, which accelerates tire production and increases environmental pollutant emissions. It is estimated that by 2030, 1.2 billion tires will be discarded annually worldwide [9,10]. In addition to ending up in dumps and landfills, the accumulation of discarded tires promotes the proliferation of rodents, insects, and other animals, causing health problems [11]. This environmental and health issue underscores the need to explore recycled tire rubber as an innovative and sustainable solution in developing seismic isolators, potentially replacing all or part of the natural elastomer matrix [4,12,13,14,15,16]. However, it is important to note that using recycled rubber from vehicle tires in seismic isolators is still in the research phase, highlighting the ongoing need for studies and tests to evaluate its effectiveness and safety in seismic applications.

To analyze recycled tire rubber as a matrix for seismic isolators, it is essential to consider the various methods employed by researchers to obtain the material, including shredded and unshredded forms, as well as the construction processes of the devices [13,15]. Beyond mechanical shredding, other tire recycling techniques include pyrolysis, thermolysis, and cryogenic shredding [11].

The use of recycled tire rubber in the manufacture of fiber-reinforced elastomeric isolators (FREIs) represents a cost-effective alternative. Cilento et al. [4] developed a reclaimed-rubber compound (RC) for recycled-unbonded FREIs (RU-FREIs), that is, a combination of recycled tire rubber granules and virgin rubber in a 1:1 ratio, supplemented with additives for re-vulcanization. Their findings indicate a 55% reduction in RC costs compared to pure natural rubber, with the material achieving shear deformations up to 150% and a cushioning capacity of up to 19% [4]. Further experimental tests have been conducted on prototypes with matrices composed entirely of recycled rubber and polyurethane binders. Calabrese et al. [17] assessed bilinear hysteresis and Bouc-Wen models to predict the seismic response of buildings isolated using fiber-reinforced recycled rubber matrix composite devices. They derived model parameters through mechanical characterization of low-cost prototypes and validated these models against shaking table tests to predict maximum structural responses accurately. In Colombia, Ortega et al. [14] developed seismic isolator prototypes with a granular matrix of recycled tire rubber, bonded with polyurethane binders and reinforced with polyester fibers. Shear tests indicated that a density of 0.99 g/cm^3^ yielded a lower shear modulus (G) compared to a density of 1.24 g/cm^3^. Other researchers have proposed isolators made of square layers of unbonded scrap tire isolator (U-STI) sheets adhered with cyanoacrylate-based adhesives. Shaking table tests revealed a 40% decrease in effective horizontal stiffness as the amplitude of horizontal displacement increased from 10 mm to 40 mm, while the isolators maintained stability under overturning deformation, returning to their original geometry post-test [13,15].

With the aim to ensure efficient seismic performance in isolators utilizing rubber, it is essential to account for both the viscosity and great elasticity components of the material, it is mean the viscoelasticity behavior. Viscoelasticity component enables the material to dissipate energy through its viscous nature, which is necessary for vibration damping and the attenuation of seismic movements. On the other hand, elasticity component is important for withstanding large deformations without sustaining permanent damage, ensuring that the material can return to its original shape after the application of extreme loads and with a nonlinear behavior [6]. These two behaviors are complementary and must be considered together in the design of isolators. The viscosity model provides energy dissipation capacity, while the elastic model ensures resistance and durability against significant deformations [6]. Analyzing the viscoelastic behavior of polymers, in some cases, such as resins, the trend at different frequencies is that the elastic component is lower than the viscous component [18]. In other polymers, like the tire rubbers, the elastic component dominates as the load is applied with low frequencies [19].

The drawbacks associated with a matrix incorporating recycled rubber include limitations in elongation and tensile strength at failure, as it is a composite material and does not exhibit monolithic characteristics. These particularities pose significant challenges to its suitability for producing conventional isolators, particularly when maximum shear deformations of up to 300% are required [17]. Nevertheless, this matrix must comply with the mechanical specifications for seismic isolation [20], ensuring that it provides the isolator with homogeneity, low horizontal stiffness, and the required damping properties.

This paper presents the development of a matrix composed of recycled rubber (RR) from vehicle tires, intended for use in seismic isolators of the type RR-FREIs for low-rise buildings. The particles were bonded using an MDI prepolymer binder (PB-MDI). The matrices were manufactured with various particle sizes to assess whether certain grain size distributions yield better seismic response characteristics. Additionally, a calcium lignosulfonate additive and cellulose fibers were tested to enhance the uniformity of the matrix. The matrix underwent experimental evaluation through mechanical hardness, tension, compression, and cyclic shear tests. Subsequently, the selected matrix was validated using reduced-scale prototypes subjected to cyclic compression and cyclic shear tests. For comparison, prototypes were also constructed using a recycled rubber matrix developed by Madera Sierra et al. [21].

The abbreviations are presented in Table 1.

## 2. Theoretical Formulation of Isolators

This section aims to provide a theoretical framework for understanding and calculating the properties of seismic isolators and their components, such as the matrix and reinforcement. The presented formulas are employed to determine several critical mechanical properties essential for evaluating the effectiveness and performance of isolators. These properties include vertical and horizontal stiffness, which dictate the isolator’s ability to resist deformations under applied loads in both directions. Additionally, the shear modulus and rubber damping are examined, the latter being determined from load-displacement hysteresis curves obtained through cyclic shear tests under constant axial compression [8,14]. The vertical stiffness of a seismic isolator must be sufficiently high to minimize vertical deformations caused by the structure’s weight.

The horizontal stiffness (*K_H_*) is calculated with Equation (1), proposed by Losanno et al. for U-FREIs [2]:

(1) $$K_{H}=\frac{G_{eff}A_{eff}}{t_{r}}$$

$K_{H}$ is computed in four steps:

Step 1: The effective shear modulus ($G_{eff}$) is determined from the rubber material characterization curve for the expected levels of deformation.

Step 2: The height of the compressed isolator (h), defined as $h=H-U_{th}$, is estimated analytically, for which an expression was derived that considers the theoretical vertical displacement under the design load $\left(U_{th}\right)$ as the sum of the displacement of the unconfined rubber $\left(U_{r}\right)$ and the displacement of the unconfined rubber $\left(U_{rf}\right)$, i.e., $U_{th}=U_{r}-U_{rf}$.

The first part, $u_{r}$, is calculated as the product of the initial height *H* and the unit strain, ε, at the limit point of stiffness change $u_{r}=H\varepsilon$. This deformation *ε* is obtained from the result of the compression test of pure rubber. The second part, $u_{rf},$ is calculated using the expression $u_{rf}=∆P/K_{Vd}$, where ∆P is the maximum applied load (P) minus the load at the stiffness change limit $(P_{sc}$); the theoretical vertical dynamic stiffness $K_{Vd}$ is obtained with Equation (2)

Step 3: subsequently, the effective area is calculated according to the deformation level. For this purpose, the method of Russo et al. was modified to be applied to a circular isolator. As a result, the portion of the isolator in contact with the supports and subject to pure shear $\left(A_{eff}\right)$ will be equal to the total area (*A*) minus the area of the detached semicircle ($A_{d}$) [2]. The detached area is calculated as $A_{d}=R^{2}/2(\theta -\sin\theta )$, with $\theta =2\sin^{-1}\left(c/2R\right)$ and length $c=\sqrt{(R-s/2)8s}$. The separation point ($d_{0}$) and the length of the detached portion (*s*) are calculated as $d_{0}=\sqrt{H^{2}-h^{2}}$ and *s* = d-d_0, respectively. Based on $d_{0}$ and the displacement level (*d*), $A_{eff}$ calculated as Equation (2).

(2) $$\left\{\begin{array}{c} A_{eff}=A,\;for\;d\leq d_{0}\;\\ A_{eff}=A-A_{d},\;for\;d>d_{0} \end{array}\right.$$

Step 4: finally, *KH* is calculated for different strain levels with the functions obtained from *G*_*eff*_ and *A*_*eff*_.

Where *G_eff_* is the effective shear modulus of the rubber, which is obtained from the characterization curve for the expected deformation levels. *A_eff_* is the effective contact area (the portion of the isolator in contact with the supports and subject to pure shear), considering the influence of the vertical behavior in the determination of the lateral displacement. *t_r_* is the height of the rubber layer.

The vertical stiffness (*K_V_*) is calculated according to Equation (3) [7]. The compressive modulus for the stiffening (*E^f^*_*c*_) is obtained from the calculation of the parameters *α* (Equation (4)) and *β* (Equation (5))

(3) $$K_{V}=\frac{E_{c}^{f}A}{H_{r}}$$

where *E*^*f*^_*c*_ is the compressive modulus for flexible reinforcement, *A* is the total cross-sectional area of the isolator, *H_r_* is the total height of the rubber given by

(4) $$\alpha^{2}=\frac{12\left(1-v^{2}\right)G_{20}R^{2}}{\left(E_{f}t_{f}t_{r}\right)}$$

where *v* is the Poisson’s ratio of the fiber, G_20_ is the shear modulus at 20% strain, *R* is the radius of the isolator, *E_f_* the tensile elastic modulus in the reinforcing fiber, *t_f_* the fiber thickness and *t_r_* the thickness of each layer of rubber.

(5) $$\beta =\frac{12G_{20}R^{2}}{Kt_{r}}$$

Here, G_20_ is the shear modulus at 20% strain, *R* the isolator radius, *K* the compressibility factor and *t_r_* the thickness of each rubber layer.

The shear modulus (*G*) is determined using the relation

(6) $$G=\frac{\tau_{max}-\tau_{min}}{\gamma_{smax}-\gamma_{smin}}$$

where *γ*_*smax*_ and *γ*_*smin*_ are the maximum and minimum shear strains of the hysteresis cycles; *τ*_*max*_ and *τ*_*min*_ are the maximum and minimum shear stresses (*τ* = *F*/*A*); *F* is the measured force, and *A* is the shear area of the specimen [22].

The damping ratio (*β*) of the rubber is calculated using [23]:

(7) $$\beta =\frac{W_{d}}{2\pi K_{eff}{{∆}^{2}}_{max}}$$

where *W_d_* is the energy dissipated in each cycle, *K_eff_* is the effective horizontal stiffness (Equation (7)) and Δ*_max_* is the average of the maximum positive and negative and negative displacements, as

(8) $$K_{eff}=\frac{F_{max}-F_{min}}{u_{max}-u_{min}}$$

where *F_max_* and *F_min_* are the forces at maximum and minimum deformation respectively, *u_max_* and *u_min_* are the maximum and minimum deformation respectively.

## 3. Materials and Methods

The RR-FREI type seismic isolator prototypes consist of a matrix made from ground tire rubber (GTR) particles, which are bonded together using a binder. This matrix is reinforced with fibers and an adhesive, ensuring the cohesion between the fibers and the GTR layers. Additionally, calcium lignosulfonate was incorporated as an additive, and cellulose fibers were included to enhance the matrix’s properties. A detailed description of these components is provided below:

### 3.1. Materials

#### 3.1.1. Ground Tire Rubber (GTR)

**Figure 1:**
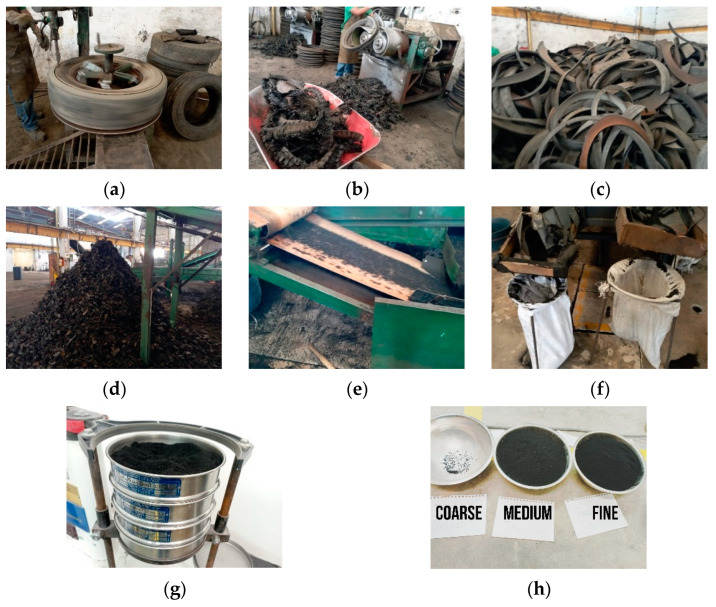
Vehicle tire recycling process: (**a**) Tire selection and sidewall cutting, (**b**) Bead cutting process, (**c**) read cut into strips, (**d**) Initial shredding, (**e**) Milling and removal of remaining steel, (**f**) Initial sifting and packaging, (**g**) mechanical sifting and (**h**) Particle size distribution.

In the initial phase of this research, ground tire rubber (GTR) was sourced from the Occidental de Cauchos production plant located in Cali, Colombia, where a stringent recycling process is implemented. This facility specializes in the collection and processing of used tires, ranging from 17.5 to 22.5-inch rims, typically used in buses and tractor-trailers. The detailed recycling procedures undertaken at this plant are depicted in Figure 1. Subsequently, at the Laboratorio de Mezclas of the Pontificia Universidad Javeriana Cali, the GTR underwent mechanical sifting to classify it into smaller particle sizes: fine, medium, and coarse (as illustrated in Figure 1g,h). The particle size distribution is provided in Table 2.

#### 3.1.2. Binder

An MDI prepolymer binder (PB-MDI) was employed to bond the GTR particles, chosen for its high elongation and adhesion strength. This binder contains free isocyanate groups that react with moisture, necessitating the immersion of specimens in water to expedite the curing process.

#### 3.1.3. Additions to GTR Matrix

**Figure 2:**
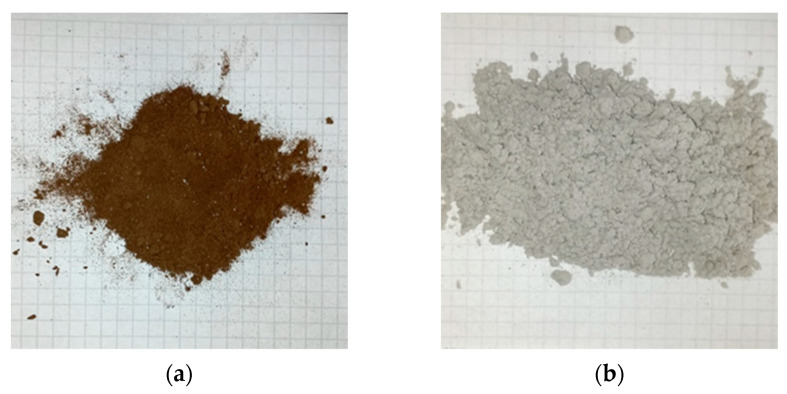
Additions to GTR matrix: (**a**) Calcium lignosulfonate and (**b**) Cellulose fibers.

A calcium lignosulfonate additive (Figure 2a) and cellulose fibers (Figure 2b) were incorporated in small percentages relative to the total weight of the GTR. The calcium lignosulfonate additive, a powder derived from sugarcane bagasse, was utilized to enhance the homogeneity of the matrix between the GTR and the binder, aiming to fill the voids within the matrix. Additionally, given that agglomerated GTR tends to detach under tensile and shear stresses, cellulose fibers were evaluated as micro-reinforcement to mitigate this issue.

#### 3.1.4. Reinforcement

**Figure 3:**
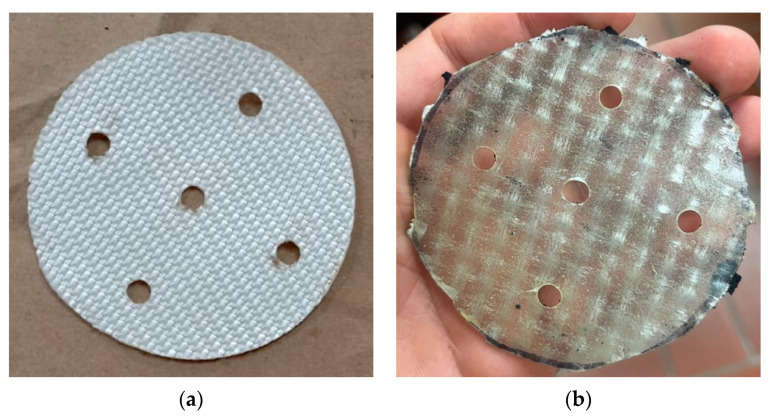
Flexible reinforcement: (**a**) Bidirectional polyester [24] and (**b**) Glass fiber mesh with epoxy resin [22].

To validate the matrix developed in this study, the second phase consisted of testing prototype RR-FREIs, which use flexible reinforcement. For this purpose, two fibers were tested as reinforcement, the first one of bidirectional polyester (Figure 3a) evaluated by Losanno et al. [24] and the second one was Glass fiber mesh with epoxy resin (Figure 3b) developed by Rivas et al. [25]. The mechanical properties of the fibers are shown in Table 3.

To validate the matrix developed in this study, the second phase involved testing prototype RR-FREIs that incorporate flexible reinforcement. Two types of fibers were tested as reinforcement materials. The first fiber was a bidirectional polyester (Figure 3a), previously evaluated by Losanno et al. [24], while the second was a glass fiber mesh with epoxy resin (Figure 3b), developed by Rivas et al. [25]. The mechanical properties of these fibers are detailed in Table 3.

#### 3.1.5. Adhesive for Interface Recycled Rubber-Reinforcement

Given that GTR particles do not undergo a revulcanization process, it is important to utilize an adhesive that ensures a robust bond at the interface between the rubber layers and the reinforcement. In this study on the fabrication of seismic isolator prototypes, two types of adhesives were employed. The first adhesive was the MDI prepolymer binder (PB-MDI), initially used for bonding GTR particles, and the second was a hybrid mounting adhesive (PF). The mechanical properties of these adhesives, as determined by Rivas et al. [25], are presented in Table 4.

### 3.2. Experimental Program

This research involved the analysis of 10 different matrices through comprehensive mechanical testing. The matrix demonstrating the most favorable mechanical properties was then selected for the fabrication of seismic isolator prototypes. These prototypes were subsequently subjected to mechanical tests to assess their performance.

#### 3.2.1. Experimental Program for Matrices of Seismic Isolators

Ten matrices, as listed in Table 5, were evaluated through mechanical tests conducted in accordance with current standards. The fabrication process of the samples was consistent across all matrices, beginning with the weighing of materials, followed by mixing, molding the material into metal molds shaped according to the specimen requirements specified by the standards, pressing into a layer using a hydraulic press at 140 °C, and subsequent curing through water immersion. These samples were fabricated at the Seismic and Structural Engineering Research Laboratory of the Pontificia Universidad Javeriana Cali.

Initially, matrices were fabricated using GTR and PB-MDI. Based on the supplier’s recommendation, an initial binder dosage of 15% by weight relative to the total weight of GTR was selected. These matrices were produced with varying particle size distributions, identified as Matrices M1, M2, M3, M4, and M5. The specific particle size distributions for each matrix are detailed in columns 2, 3, and 4 of Table 5.

After evaluating the different particle sizes, it was observed that the M4 matrix exhibited the best performance. Consequently, the percentage of PB-MDI binder was adjusted, leading to the development of matrices M6 and M7, which utilized 20% and 10% binder, respectively, as detailed in column 5 of Table 5.

Further evaluation involved the inclusion of additives such as calcium lignosulfonate (column 7 of Table 5) and cellulose fibers (column 8 of Table 5) in small percentages. Specimens were manufactured using the previously selected particle size distribution while maintaining a 15% PB-MDI binder content. These new matrices were designated as M8, M9, and M10.

Following the development of the matrices, the mechanical tests performed are detailed below. These tests are essential for evaluating the behavior and properties of each matrix. Hardness and cyclic shear tests were conducted on all matrices. Subsequently, the matrix that demonstrated the best performance in the shear test was selected for additional testing, including monotonic compression, residual compression, and tension tests. These tests are necessary for providing comprehensive insights into the mechanical performance of the matrices under various loading conditions. The tension, monotonic compression, and cyclic shear tests were conducted using the INSTRON 3366 universal testing machine, which has a capacity of 10 kN and is located at the Laboratorio de Materiales y Procesos at Pontificia Univrsidad Javeriana Cali.

**Figure 4:**
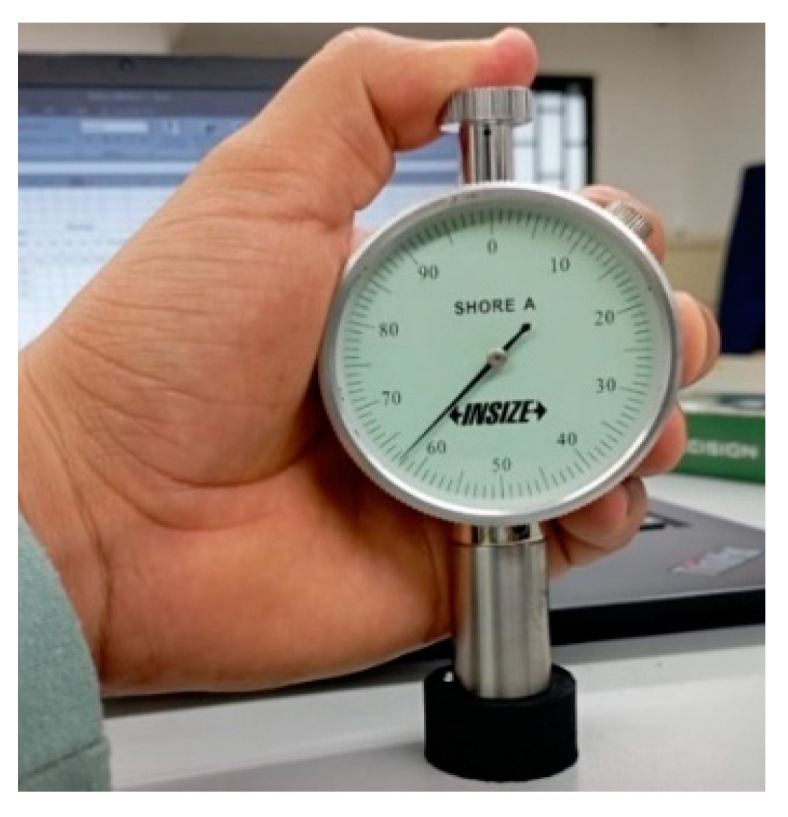
Matrix hardness test.

The hardness of the matrix was determined in accordance with ASTM D2240 [26] using the Shore A durometer at the Materials and Processes Laboratory of PUJ Cali (Figure 4). This test measures the penetration resistance on the Shore A scale, which is suitable for softer materials. For each matrix, three specimens were fabricated, and five measurements were taken for each specimen. The specimens had a cylindrical geometry with a diameter of 28.6 mm and a height of 12.5 mm. A minimum of five readings were taken per specimen, with each reading separated by at least 6.00 mm (0.24 in).

**Figure 5:**
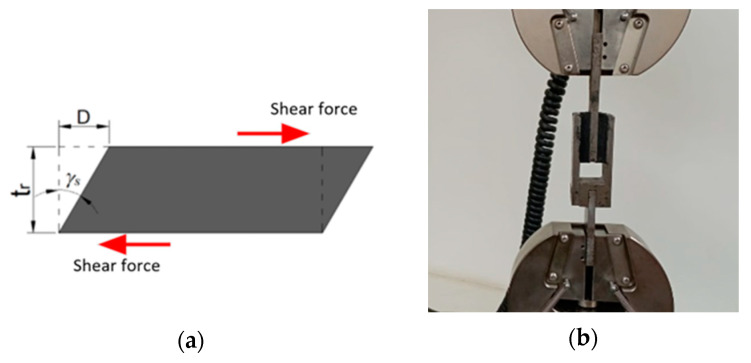
Matrix cyclic shear test (**a**) Shear deformed specimen and (**b**) Assembly.

The cyclic shear test was conducted in accordance with Method B of ASTM D4014 [27], using the setup illustrated in Figure 5b. The test specimens consisted of rectangular rubber sheets measuring 40 mm in length, 30 mm in width, and 7.5 mm in thickness, which were manufactured separately and bonded using epoxy. Three specimens from each matrix were tested. The test involved applying 10 loading and unloading cycles at varying deformations (γs) of 10%, 20%, 30%, 50%, 75%, and 100%, period of 9s (maximum of machine), by adjusting the maximum displacement D (Figure 5a). The tests were carried out using an INSTRON 3366 universal testing machine with capacity of 10 kN, located in Laboratorio Materiales y Procesos at PUJ Cali (Figure 5b). These deformation percentages correspond to the maximum strains that an isolator might experience during operation. The chosen deformation values align with FEMA guidelines, which recommend that isolators be tested prior to installation under bidirectional loads, with deformations exceeding (a) 25%, (b) 50%, (c) 67%, and (d) 100% of design deformation [23,28].

**Figure 6:**
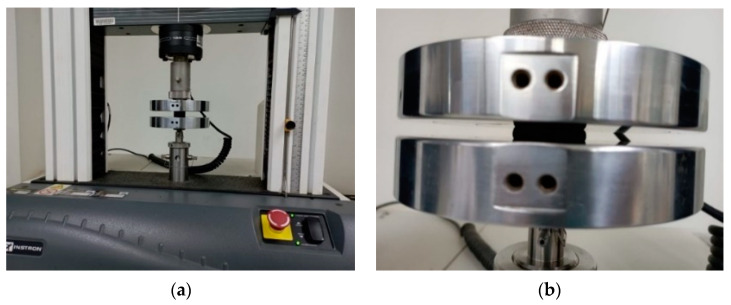
Matrix monotonic Compression Test (**a**) Assembly and (**b**) Specimen deformed by compressive loading.

A monotonic compression test was conducted to assess the compressive strength of the matrix, as illustrated in Figure 6a. The specimens used were cylindrical, with a diameter of 28.6 ± 0.1 mm and a height of 12.5 ± 0.3 mm, in accordance with ASTM D575 Method A [29]. Compressive stress at specified strain levels was measured at a rate of 12 ± 3 mm/min. The testing procedure included three consecutive loading and unloading cycles, with the initial two cycles serving to condition the specimens up to 40% strain. Measurements were taken during the third cycle. The final loading cycle continued until the equipment reached its maximum deformation capacity, which is slightly less than 80% strain (see Figure 6b). Principio del formulario

Final del formulario

**Figure 7:**
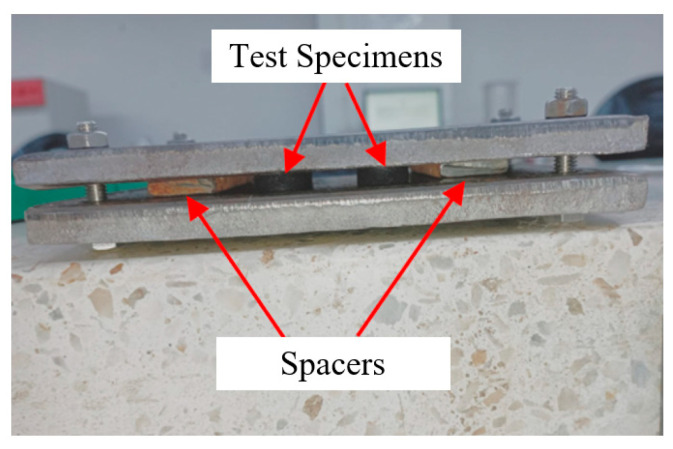
Residual compression test set-up.

The residual compression test was performed in accordance with Method B of ASTM D395 [30]. This test measures the deformation of a specimen after exposure to a constant compressive load under elevated temperature conditions. Cylindrical specimens were placed between steel plates with a spacer, allowing for a 25% deformation when secured with bolts, as illustrated in Figure 7. The assembly was then placed in an oven at 70 °C for 22 h. After this period, the specimens were removed from the assembly and left to rest at room temperature on a low thermal conductivity surface, such as wood, for 30 min. The final specimen height was subsequently measured to determine the residual compression, using the formula *CB* = [(*t*_0_ – *t*_*i*_)/(*t*_0_ – *t*_*n*_)] × 100, where *t*_0_ is the original specimen thickness, *t*_*i*_ is the final specimen thickness, and *t*_*n*_ is the thickness of the spacer.

**Figure 8:**
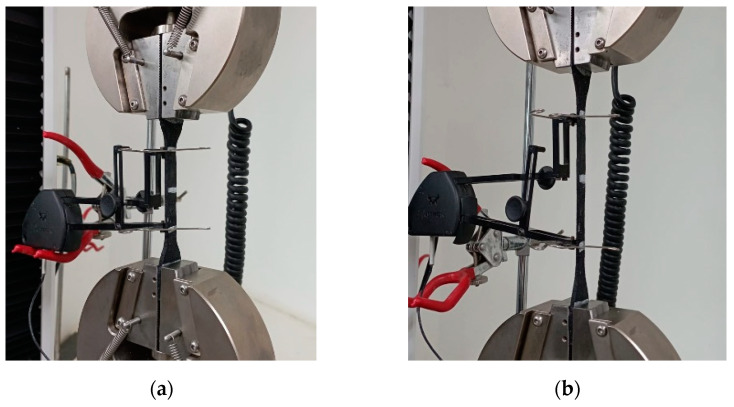
Matrix tensile Test: (**a**) Undeformed specimen and (**b**) Deformed specimen.

The tensile strength of the recycled rubber was evaluated according to the procedure outlined in ASTM D412 [31]. As depicted in Figure 8, dumbbell-shaped specimens were clamped at both ends using grips and elongated until rupture. The test measured tensile stress, tensile strength, and the maximum yield point, with the elongation rate set at 50 ± 5 mm/min. Strain measurements were obtained using an extensometer.

#### 3.2.2. Seismic Isolator Prototypes

**Figure 9:**
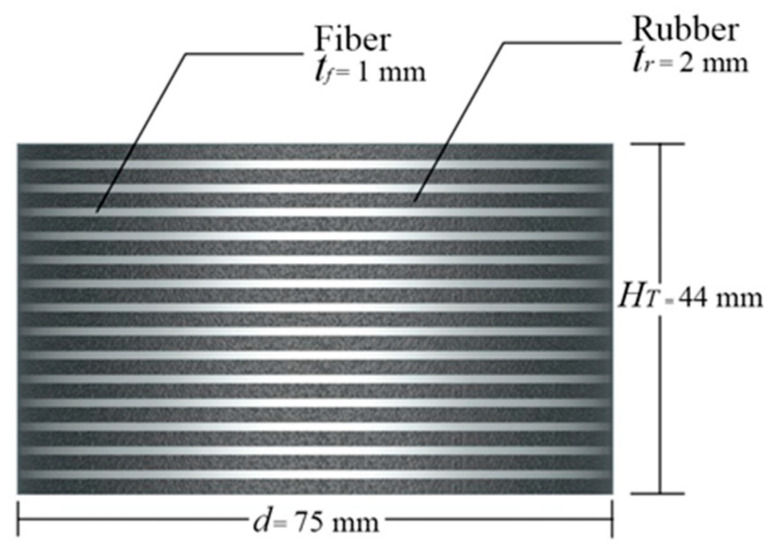
Specimen Geometry.

The prototype dimensions were derived from designs by Madera using natural rubber as a reference [2,6]. The test specimens were developed for shaking table tests on a prototype building associated with the Department of Structures for Engineering and Architecture (DiSt) at the University of Naples Federico II, Italy. The design process adhered to FEMA 450 requirements. The structure comprised a steel frame with two degrees of freedom, a total height of 2900 mm, and plan dimensions of 2650 × 2150 mm. The structure’s total mass was 77 kN, distributed as 36 kN at the base level and 41 kN at the top level, resulting in a load of 19 kN at the base of each column. The RR-FREI prototype was designed with a diameter *d* = 75 mm and a total height *H_T_* = 44 mm. These prototypes feature alternating layers of fiber reinforcement and rubber, specifically 15 layers of rubber *n_r_* = 15 and 14 layers of fiber *n_f_* = 14 as illustrated in Figure 9.

**Figure 10:**
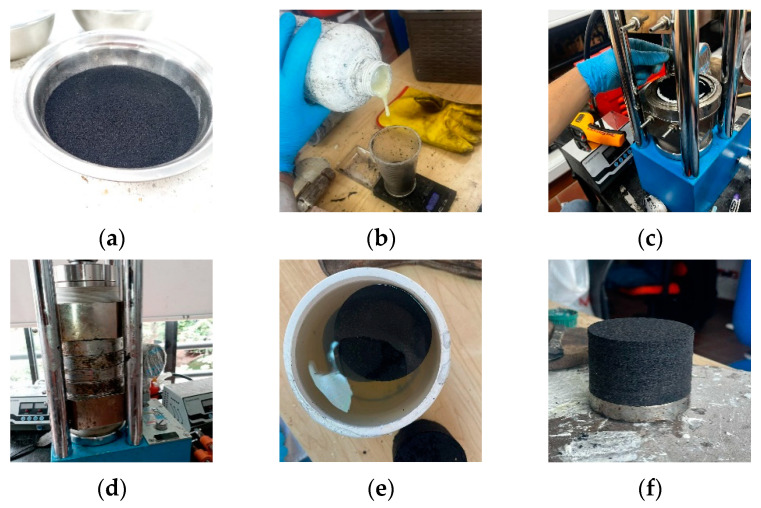
Prototype manufacturing process: (**a**,**b**) material weighing, (**c**) molding, (**d**) pressing, (**e**) curing process and (**f**) finished prototype.

The prototypes were fabricated at the Seismic and Structural Engineering Research Laboratory of the Pontificia Universidad Javeriana Cali. As detailed in Figure 10, the process began with the precise measurement and mixing of crushed rubber with the binder. The mixture was then placed into a stainless-steel mold in alternating layers of rubber and reinforcement. This assembly was subjected to pressing at 140 °C for 40 min. The application of heat was necessary to ensure adhesion between the particles, as the binder used is not effective at low temperatures. The process was completed with an 8-h water curing period, ensuring proper setting and durability of the prototypes.

Prototypes of RR-FREIs were primarily manufactured using the M4 matrix, which had previously exhibited optimal mechanical performance in earlier testing phases. To investigate the impact of binder content, additional prototypes were produced using the M7 matrix, which contained 10% binder. Regarding the reinforcement layers applied between the rubber layers, two options, as outlined in Table 3 were evaluated. Adhesion between the reinforcement layers and the rubber was achieved using the adhesives specified in Table 4. The various prototype configurations are detailed in Table 6.

Cyclic compression and cyclic shear tests were conducted on one specimen for each test and variation to assess the mechanical properties of the prototypes and to validate the final matrix composition. The specific procedures for each of these tests are described in the following sections.

**Figure 11:**
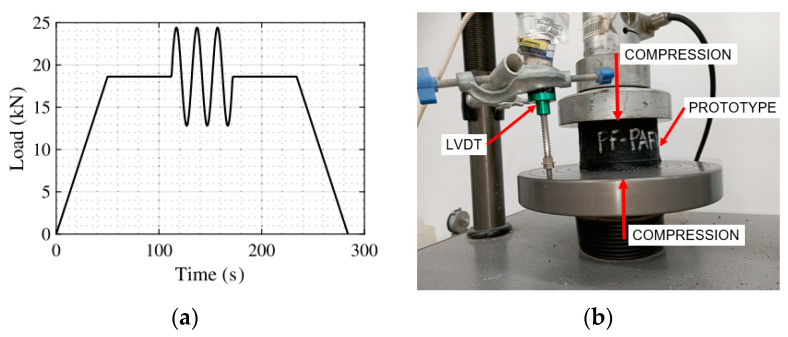
Cyclic compression test: (**a**) Protocol and (**b**) Compression test setup.

A cyclic compression test was conducted at the Mixing Laboratory of Pontificia Universidad Javeriana Cali to evaluate the mechanical response of the prototypes to compression loads and to determine their experimental vertical stiffness (Kvdex). The test utilized a Geotest Instrument Corp machine with a compression capacity of 5 tons. Displacements during the test were accurately measured using a linear variable differential transformer (LVDT), as shown in Figure 11b.

The test was conducted in three stages, following the methodology outlined by Losanno et al. [3,4]. Initially, a preload was applied to the prototypes and incrementally increased at a rate of 0.01 mm/s until the design load of 19 kN was achieved. Upon reaching this load, the prototypes were allowed to rest for 1 min to ensure stabilization. Subsequently, three consecutive triangular load-unload cycles were performed, varying by ±30% relative to the design load, at a speed of 0.05 mm/s. After the final cycle, the specimens were allowed to rest for an additional minute before being fully unloaded at 0.01 mm/s. This controlled testing procedure effectively characterizes the cyclic behavior of the prototypes under analysis.

The experimental vertical stiffness (Kvdexp) is determined from the force-displacement curve generated by the test equipment. It is calculated as the slope of the linear segment passing through the cyclic portion of the curve [2].

**Figure 12:**
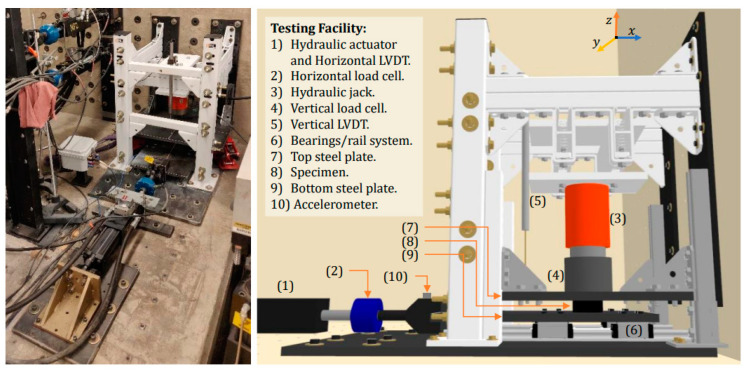
Cyclic shear test setup [32].

Cyclic shear tests were conducted using the testing frame at the Intelligent Infrastructure Systems Laboratory (IISL) at Purdue University (Figure 12) to evaluate the horizontal response of the seismic isolator prototypes. This moment-resisting steel frame, mounted on a concrete slab, is designed to apply a vertical compressive force of up to 22 kN on the prototype—simulating the weight of the superstructure—using a hydraulic jack. Simultaneously, a variable horizontal force of ±8.8 kN was applied by a hydraulic actuator. This setup allowed the prototypes to be subjected to a constant vertical force and a variable horizontal force, effectively simulating the conditions experienced during a seismic event.

**Figure 13:**
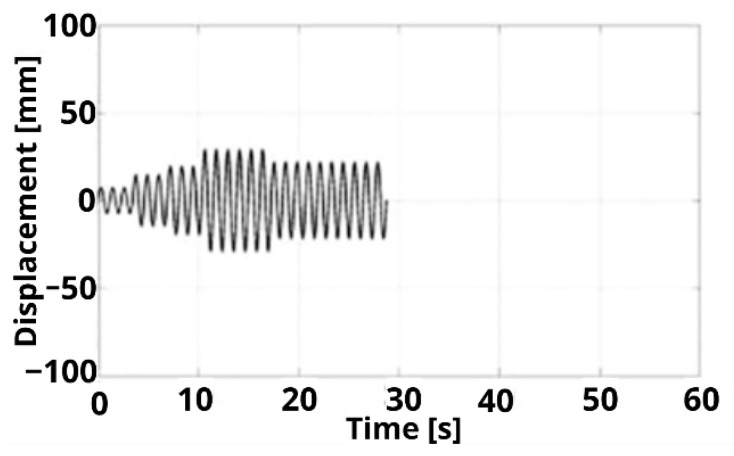
Protocol cyclic shear test.

The test protocol involved applying six levels of controlled horizontal deformation, following the recommendations of Madera I. [22]. The deformation levels corresponded to 25%, 50%, 67%, 100%, 100%, and 75% of the selected maximum deformation of 29 mm for the prototypes, with a period of 1.15 s, as illustrated in Figure 13. By systematically increasing the horizontal displacement in controlled cycles, this test effectively characterized the cyclic shear behavior of the seismic isolator prototypes under analysis.

## 4. Results and Discussion

This section presents the results of the various mechanical tests conducted, accompanied by a detailed analysis of the evaluated matrices and prototypes. To ensure accurate interpretation, these results are compared with findings from previous studies by other researchers, both for the matrices and the isolator prototypes. This comparison is necessary for verifying the outcomes, assessing their mechanical properties, and evaluating their validity in relation to the current state of knowledge in the field.

### 4.1. Matrices for Seismic Isolators

Hardness and cyclic shear tests were conducted for each of the matrices. Based on the performance in the cyclic shear test, the matrix that exhibited the best performance was selected for further testing. The results were then compared with those obtained by Madera using a natural rubber matrix [6]. Additionally, comparisons were made with the results of tests performed under the same conditions as those used by Madera et al. [21,28], but utilizing recycled rubber.

#### 4.1.1. Hardness Test

Table 7 presents the hardness results for specimens M1, M2, M3, M4, and M5, which were fabricated using matrices with varying particle sizes. The data indicate that an increase in the concentration of medium particle size correlates with higher hardness values. When examining specimens M4, M6, and M7, it is evident that matrices with a higher binder percentage also exhibit increased hardness. Notably, the M8 matrix, which incorporated calcium lignosulfonate, demonstrated the lowest hardness, closely approximating that of natural rubber [6]. Conversely, the M9 and M10 matrices, which included cellulose fiber, displayed the highest hardness values, with this property intensifying as the fiber concentration increased. The matrices exhibit a low standard deviation, ranging from 0.3 to 1.3, indicating that the hardness properties are consistent and stable across different samples.

All matrices using recycled rubber exhibited greater hardness compared to the natural rubber matrix developed by Madera et al. [6], though they had lower hardness than those using recycled rubber as reported by Madera et al. [21]. Importantly, all hardness values were considered acceptable, as they fell within the typical range of 40–70 A, which is standard for rubbers used in seismic isolation applications [33].

#### 4.1.2. Cyclic Shear Test

The shear modulus (G) and damping ratio (*β*) were determined from the cyclic tests detailed in Section 2, using Equations (6) and (7), respectively. The results for the various matrices are reported at 20% deformation, a representative value commonly used in isolator design [6], as well as at 50% deformation for comparison with other authors’ findings [21,28].

Table 8 indicates minimal variation in the results across matrices M1, M2, M3, M4, and M5. However, certain trends are noteworthy: matrices M1, composed entirely of fine material, and M5, composed entirely of medium material, exhibited higher shear moduli. Additionally, at 20% deformation, these matrices demonstrated lower damping ratios compared to the others. Matrix M3, which contains a 50% mixture of fine and medium materials, also showed a high shear modulus (G), but it was observed that at 50% deformation, this matrix allowed for higher damping. Among the matrices with lower shear modulus, M2 and M4, was selected for further investigation due to its lower fine material content, which simplifies the sieving process and material preparation.

Binder variation was conducted using the particle size distribution of the M4 matrix. The results obtained from matrices M4, M6, and M7 demonstrated a direct correlation between an increased binder percentage and an elevated shear modulus, indicating a stiffer behavior in samples with higher binder concentrations. Additionally, it was observed that samples with a lower binder proportion exhibited a greater tendency for particle detachment, despite having lower stiffness. Furthermore, an increase in binder content was associated with enhanced damping capacity. This phenomenon is likely due to improved cohesion between particles, allowing for greater deformation before failure and, consequently, more effective energy dissipation.

The addition of calcium lignosulfonate powder revealed that, upon contact with moisture, the powder developed a viscous consistency. However, when incorporated in a small proportion (3%) into the M8 matrix, a noticeable loss of interparticle adhesion occurred. This resulted in a lower shear modulus compared to other samples, although the material still reached failure at 50% strain. Given these findings, it was decided to discontinue further variations with this additive.

In contrast, the addition of cellulose fiber resulted in a significant increase in shear modulus, as demonstrated in matrices M9 and M10. However, the damping percentage was observed to vary inversely with the amount of fiber added. Upon analyzing the failure modes of the specimens, it was evident that the matrices containing cellulose fiber exhibited a failure mode similar to that of the other matrices, indicating that the fiber did not effectively function as reinforcement to enhance deformation capacity. Consequently, cellulose fiber was not included as an additive in the final matrix.

Finally, when comparing the results obtained in this test with those of Madera et al., it was observed that the various matrices containing recycled rubber from vehicle tires were 38% to 60% stiffer and exhibited 50% less damping than those using natural rubber [6]. Additionally, they were 25% stiffer and had 25% less damping than the matrix developed by Madera et al. [21] using recycled rubber.

**Figure 14:**
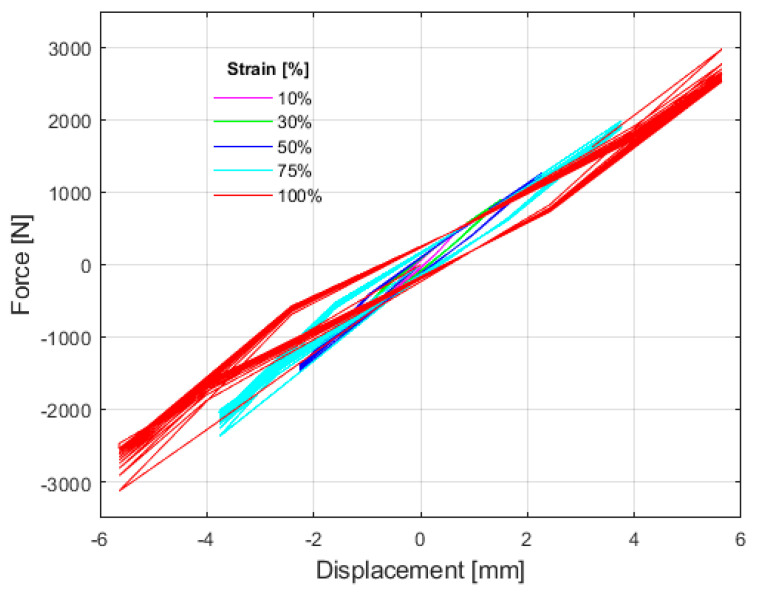
Hysteresis curve of the cyclic shear test of the M4 matrix.

Figure 14 presents the hysteresis curves of the M4 matrix, which demonstrated superior performance compared to the other matrices. The curves initially exhibit a steeper slope, indicating a higher shear modulus at the onset of deformation. As deformations increase, the curves gradually flatten, reflecting a reduction in shear modulus. The area under the curves corresponds to the energy dissipated in each cycle, suggesting that the material is stiffer at smaller deformations and becomes more flexible as the deformations increase.

**Figure 15:**
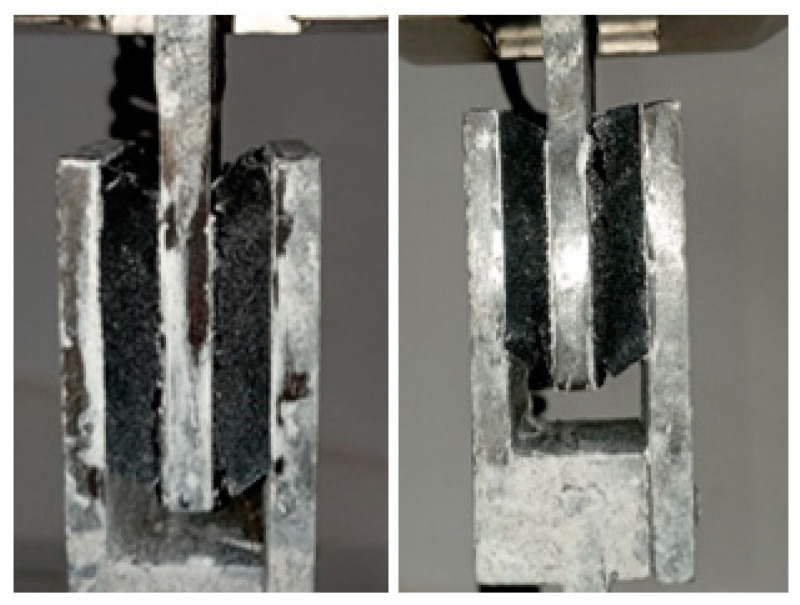
Shear failure mode.

The failure mode observed in the different matrices under shear stress is attributed to particle detachment, a result of the material’s agglomerated nature, as shown in Figure 15. All matrices failed after 50% deformation, except for M4, which failed after 100%. For this reason, M4 was selected for further mechanical testing.

The cyclic shear test was instrumental in selecting the M4 matrix for subsequent tests. This matrix, characterized by a higher proportion of medium-sized particles, offers advantages in the sieving process, as finer material requires more effort and increases production costs. M4 also has an intermediate binder content among the matrices evaluated, demonstrating superior performance in terms of stiffness and damping, despite exhibiting a tendency toward particle detachment similar to that observed in M7. While mechanical characterization continued with the M4 matrix, it was decided to also utilize the M7 matrix in prototype manufacturing. This decision aimed to evaluate the behavior with a lower binder content, given that M7 displayed properties comparable to those of the selected matrix.

#### 4.1.3. Monotonic Compression Test

**Figure 16:**
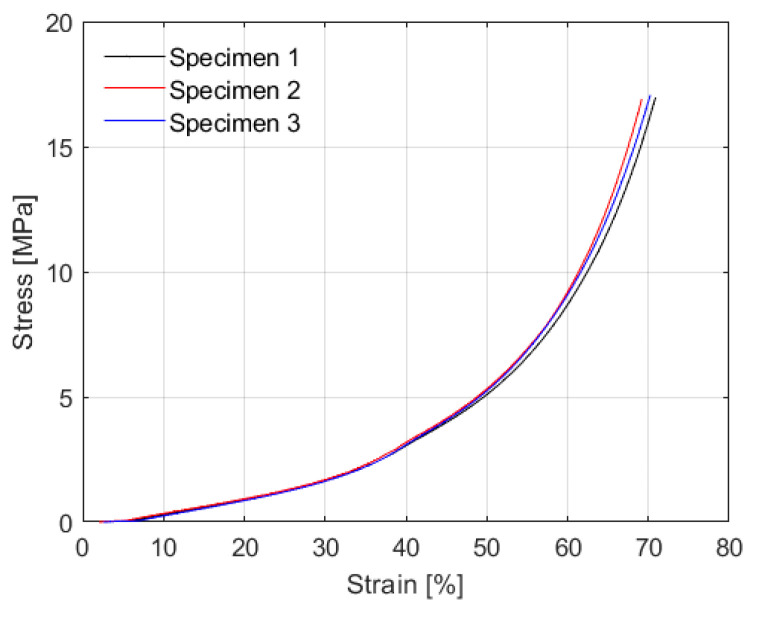
Monotonic compression test results.

The matrix evaluated, M4, is an agglomerate of rubber particles. Initially, there is an accommodation of these particles when compressive stresses are applied, allowing deformations with relatively low stresses. This phenomenon contrasts with the behavior of natural rubber, which has a more compact structure due to vulcanization, and therefore does not exhibit this initial accommodation to such an extent. Figure 16 shows the results obtained for 3 specimens tested. At 65% strain, the specimens reached an average compressive stress of 12.16 MPa and once the specimens were unloaded, the majority of the deformation (97%) was recovered. However, there is a notable difference compared to the values reported by Madera et al., who obtained 25 MPa using natural rubber [6], but with better performance than that obtained by Madera et al. with recycled rubber [21], registering a value of 6 MPa. The effects of high deformations, as well as lateral expansion due to vertical forces, can be counteracted with additional reinforcement in the design of the isolator. Such reinforcement will adequately resist the stresses transmitted by the supported structure [28,34].

#### 4.1.4. Residual Compression Test

Five specimens were fabricated from the matrix selected for this test, with the results summarized in Table 9. An average residual compression of 29.5% was obtained, which is considered acceptable since rubber used in seismic isolators typically allows a maximum residual compression of 30% [35]. This outcome suggests that the specimens tend to retain their elastic properties even after being subjected to prolonged applied loads. The low residual compression indicates that the evaluated recycled rubber largely recovers its original shape after testing. This characteristic is particularly desirable in seismic isolation matrices, where the material must absorb loads and deformations during a severe earthquake and subsequently recover to be effective in future events. The five specimens show a standard deviation (SD) of 0.9%, indicating low variability in the measurements and demonstrating uniform behavior in terms of residual deformation.

#### 4.1.5. Tension Test

**Figure 17:**
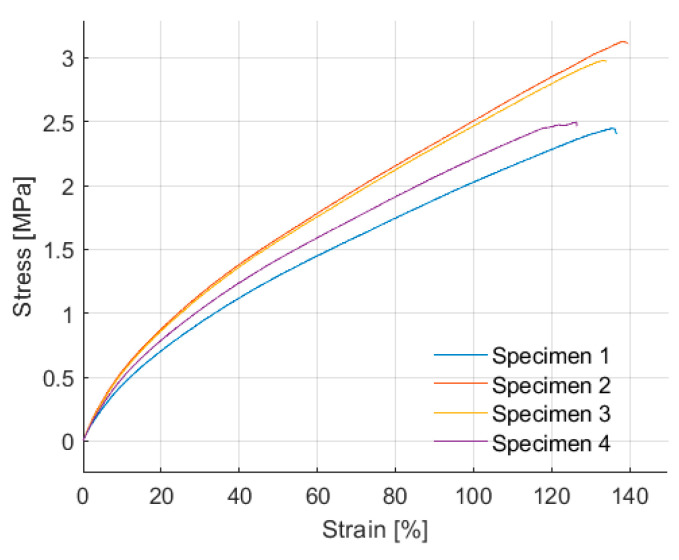
Tension test results.

Four specimens were tested under tensile stresses according to ASTM D412. As shown in Figure 17, the recycled rubber specimens reached maximum strains between 126% and 138% before failure, with tensile stresses ranging from 2.45 MPa to 3.13 MPa. These values differ significantly from the typical behavior reported for natural rubber [6], which can achieve elongations of over 500% or even 630% under tension, as summarized in Table 10. However, compared to the recycled rubber studied by Madera et al. [21], there was a 46% improvement in maximum elongation. The tension test results show moderate variability in the maximum deformation, with a standard deviation of 4.5, indicating relatively uniform behavior. Regarding the maximum tensile stress, the standard deviation of 0.3 reflects a high level of consistency in the tests. The limited extensibility of recycled rubber is attributed to its composite material nature, where the agglomerated particle matrix restricts stretching capacity. Although the recycled rubber matrix exhibits some ductility, it shows a reduced ability to withstand large elastic deformations without rupture compared to monolithic natural rubber. These mechanical properties could present a challenge in the design of seismic isolators, where high elongations are typically required [17].

The effectiveness of the water-activated polyurethane binder was also demonstrated, as it allowed the specimens to achieve greater elongation compared to those using recycled rubber as reported by other authors [21]. This indicates that the matrix with this binder exhibits more flexible behavior.

Considering that the temperature used in all the mentioned tests was ambient (27 °C), which is higher than the glass transition temperature of the analyzed matrices (−50 °C) [19], it can be confirmed that the material exhibited viscoelastic behavior [19]. Given the low frequency applied in the tensile and compressive tests (monotonic), it can be stated that the elastic component dominated in the response of the M4 matrix [19]. The opposite occurred during shear tests, where, according to the Dynamical Mechanical Analysis (DMA) carried out by other authors, as the frequency of load cycles increased, the elastic component became lower than the viscous component [36]. This behavior aligns with the requirements for the use of the M4 matrix in the manufacturing of isolators for buildings, as these devices will be subjected to static compressive loads, dead and live gravitational loads (service loads), throughout their operational life, ensuring minimal deformations over time. Furthermore, during a seismic event, the viscous component will prevail, allowing for damping of the building’s motion, thereby dissipating the energy imposed by the earthquake due to the phase lag between stress and strain. This will ultimately reduce damage to both structural and non-structural elements and minimize the loss of human lives.

### 4.2. Seismic Isolators

#### 4.2.1. Cyclic Compression

Table 11 presents the results obtained from the different prototypes. Compared to the reference prototypes made of natural rubber [2] and recycled rubber [14] developed by Ortega et al., all evaluated prototypes exhibited higher experimental vertical stiffness (Kvdexp). Specifically, the AA-PAP10 and AA-PAP15 prototypes, which contain 10% and 15% binder, respectively, and have the same reinforcement as the reference prototypes, demonstrated a vertical stiffness that was 30% higher than that of the natural rubber prototype [2] and 61% higher than that of the recycled rubber prototype [14]. This indicates that the evaluated matrix provides a stiffer response to vertical loads.

Prototypes with 15% binder exhibited higher vertical stiffness compared to those with 10%, indicating that increased binder content enhances the stiffness of the composite material. A higher binder concentration improves the cohesion between recycled rubber particles, resulting in a more compact and resilient structure. Additionally, the binder fills the spaces between particles, increasing the material’s density and improving load distribution, which contributes to greater resistance to deformation under load and, consequently, higher vertical stiffness in the prototypes with higher binder content.

Prototypes reinforced with glass fiber mesh and epoxy resin (AA-PAFV15, PF-PAFV10, PF-PAFV15) demonstrated an experimental vertical stiffness exceeding 40 kN/mm. This high stiffness ensures greater stability of the isolator under vertical loads imposed by the structure and helps prevent flaring of the device.

The difference between the theoretical stiffness (Kvdth) and the experimental stiffness ranged from 4.83% to 18.15%. These deviations are considered acceptable and are likely attributable to the variability inherent in composite materials. All prototypes exhibited low residual deformation (between 0.24% and 0.99%), indicating the recycled rubber’s ability to recover its shape after unloading.

#### 4.2.2. Cyclic Shear

**Figure 18:**
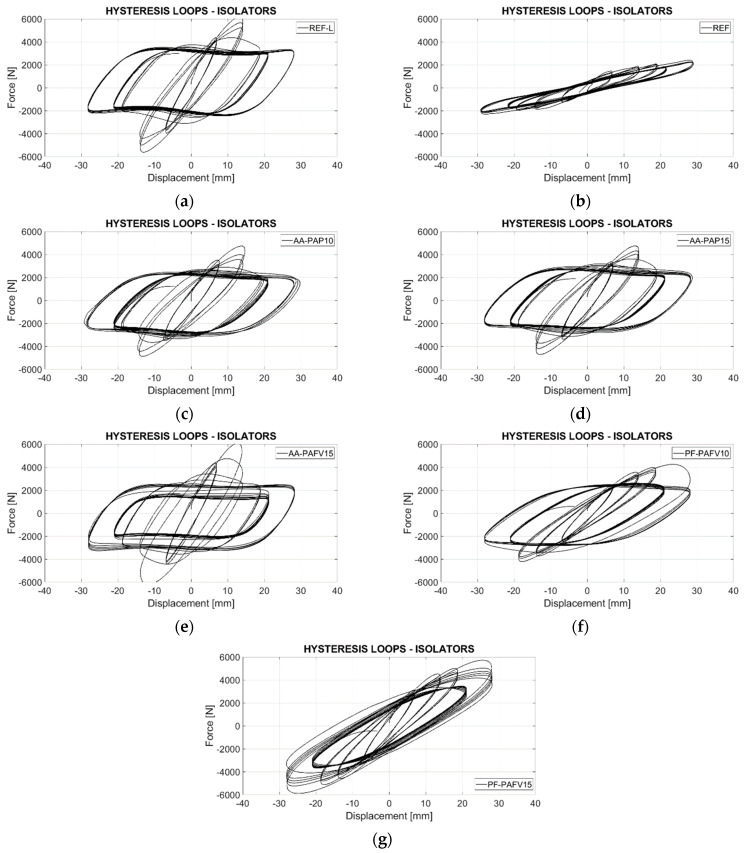
Hysteresis curves for the different prototypes: (**a**) Ref (Recycled rubber) [25], (**b**) Ref (Natural rubber), (**c**) AA-PAP10, (**d**) AA-PAP15, (**e**) AA-PAFV15, (**f**) PF-PAFV10 y (**g**) PF-PAFV15.

Figure 18 shows the hysteresis curves of the different prototypes tested, following the protocol outlined in Figure 13, which includes five levels of deformation. It is observed that the reference prototype with a recycled rubber matrix developed by Madera Sierra et al. [22] and prototypes AA-PAP10, AA-PAP15, AA-PAF15 (Figure 18a–e) failed after reaching 50% deformation, displaying instability in subsequent cycles. These prototypes exhibit a significant decrease in stiffness, as evidenced by the horizontal hysteresis curves after the third deformation cycle. The failure of these specimens is primarily attributed to base slipping after the second deformation, which prevented further deformation, with some prototypes also showing wear in the first rubber layer (Figure 20a–c). The PF-PAFV10 prototype (Figure 18f) exhibited a critical failure after reaching 67% strain, as evidenced by the horizontal hysteresis loops. This horizontal behavior in the curves indicates a severe loss in the isolator’s capacity to resist shear forces beyond this level of deformation.

**Figure 19:**
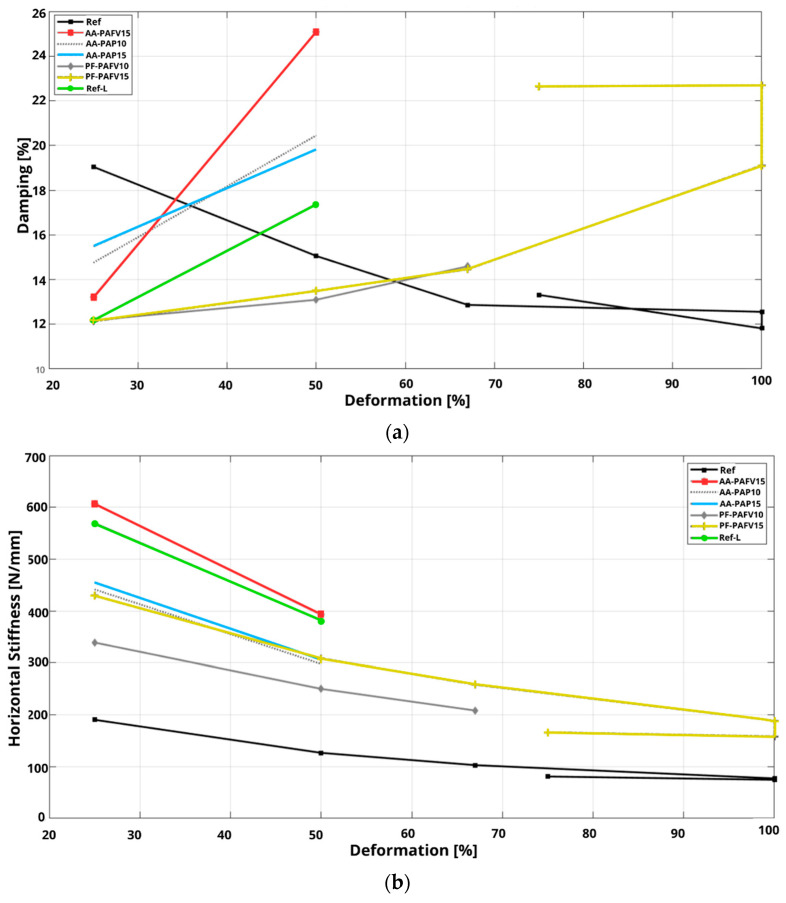
Cyclic shear test results: (**a**) Damping and (**b**) Horizontal stiffness.

**Figure 20:**
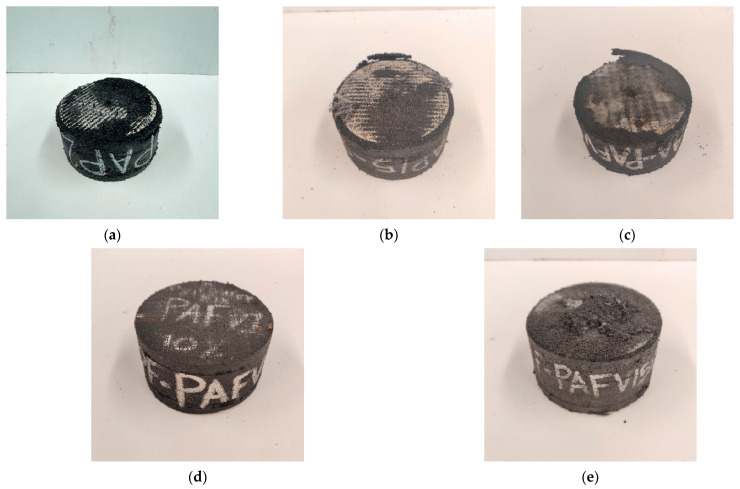
Prototypes after testing: (**a**) AA-PAP10, (**b**) AA-PAP15, (**c**) AA-PAFV15, (**d**) PF-PAFV10 y (**e**) PF-PAFV15.

The PF-PAFV15 prototype (Figure 18g) demonstrated the best performance, completing all deformation cycles without failure. Although some stiffness degradation was observed, it remained more stable than the other recycled rubber prototypes evaluated in this study. Additionally, the hysteresis curves for this prototype were wider compared to those of the reference natural rubber [2], indicating a higher energy dissipation capacity. Despite achieving higher horizontal stiffness at various deformation levels relative to the reference, its damping behavior was inversely proportional. As shown in Figure 19, the PF-PAFV15 prototype started with 12% damping at 25% deformation and increased to 22% damping at 100% deformation, unlike the reference device, which began with 19% damping and decreased to 12%. Figure 20 illustrates the condition of the prototypes after testing: AA-PAP10, AA-PAP15, and AA-PAF15 exhibited wear in the first layer, exposing the reinforcement. In contrast, PF-PAFV10 and PF-PAFV15 showed minimal wear, with the reinforcement remaining unexposed.

The importance of the surface finish of the prototypes after being subjected to cyclic shear tests with slight vertical compression lies in their ability to resist wear and protect the internal layers of the material. Devices that exhibited wear, such as the AA-PAP10, AA-PAP15, and AA-PAF15 prototypes, showed reduced durability and increased vulnerability to structural degradation, which could compromise their long-term effectiveness in seismic applications. In contrast, the PF-PAFV10 and PF-PAFV15 prototypes, which exhibited minimal wear and maintained reinforcement without exposure, demonstrate greater resistance to deterioration. This is necessary for ensuring the longevity and reliability of the isolators under real operational conditions. Maintaining structural integrity and damping capacity over time, particularly under cyclic loads and severe conditions, is essential for the effectiveness of these devices.

Table 12 summarizes the results obtained, revealing that the PF-PAFV15 prototype was the only device among those evaluated to complete all cycles at every deformation level. Compared to the reference prototype with a recycled rubber matrix [21], which only reached 50% deformation, the PF-PAFV15 exhibited 20% higher horizontal stiffness and greater damping capacity. When compared to the reference prototype using a natural rubber matrix [2], the PF-PAFV15 also demonstrated greater horizontal stiffness while achieving damping percentages that were 44% higher at 100% deformation. This enhanced damping is an essential property for dissipating energy during an earthquake and minimizing load transfer during such events.

When compared to the reference prototype using a natural rubber matrix [2], the PF-PAFV15 also demonstrated greater horizontal stiffness while achieving damping percentages that were 44% higher at 100% deformation.

Finally, the prototype that demonstrated the best physical-mechanical characteristics was PF-PAFV15. The combination of the M4 matrix with the flexible PFV reinforcement resulted in excellent damping, exceeding the 10% requirement for low-structure isolators [22], and exhibited strong performance under vertical loads, ensuring the device’s stability and integrity under such stresses. These features would be particularly advantageous for buildings subjected to earthquakes. During a seismic event, an isolator with the characteristics of the PF-PAFV15 prototype could significantly mitigate the horizontal forces transmitted to the structure due to its superior damping capacity and resistance to vertical loads. This could substantially reduce deformations and structural damage, thereby enhancing the building’s safety and stability during such events.

**Figure 21:**
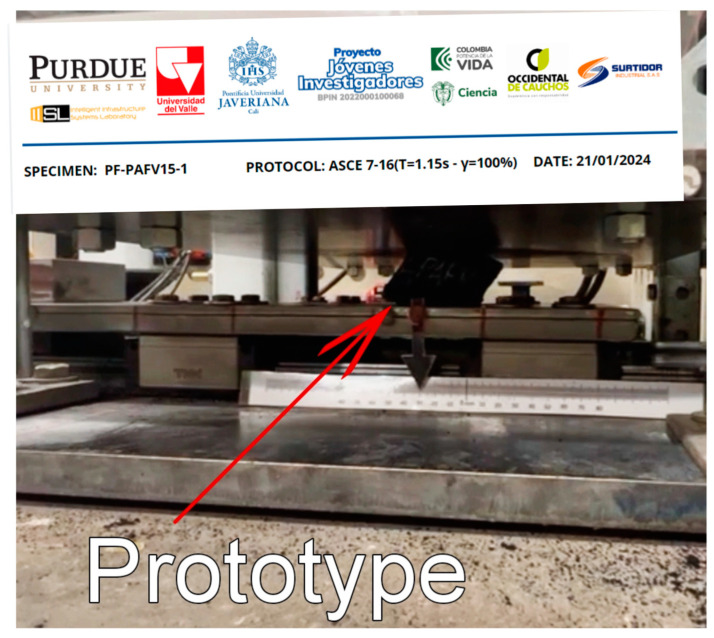
Rollover condition for the prototype PF-PAFV15.

However, due to the inherent characteristics of Recycled Rubber Fiber-Reinforced Elastomeric Isolators (RR-FREIs), the PF-PAFV15 prototype exhibited a specific phenomenon upon reaching 100% deformation—overturning. This condition, in which a portion of the upper and lower surfaces detaches from the support plates, is characteristic of these devices, as illustrated in Figure 21.

## 5. Conclusions

This article presents the development and characterization of a matrix composed of ground tire rubber particles from end-of-life vehicles, bonded with a polyurethane binder. The study focuses on the initial phase of a research project on Eco-Isolators, specifically aimed at validating the mechanical behavior of the matrix in low-cost seismic isolator prototypes of the RR-FREIs type.

Initially, various matrix configurations were evaluated through mechanical testing by adjusting particle size, binder content, and the use of additives. The hardness test results indicated that all variations remained within the appropriate range for seismic isolation applications. The cyclic shear test was important in selecting the matrices for mechanical testing of the prototypes. In this context, the M4 matrix, composed exclusively of ground tire rubber (GTR), exhibited 38% higher stiffness and 50% lower damping capacity compared to the natural rubber matrix, demonstrating the best overall performance. Additionally, it was observed that at deformations exceeding 50%, all matrices failed due to particle detachment except for the M4 matrix, which withstood deformations up to 100%. In the monotonic compression test, the M4 matrix showed 50% lower vertical stiffness than natural rubber but 50% higher than that obtained with recycled rubber reported by other authors. In the residual compression test, the M4 matrix met the required standards for seismic isolation, showing low residual compression, which indicates that the evaluated recycled rubber largely recovers its original shape after the application of a sustained load. Finally, in the tensile test, the M4 matrix exhibited maximum deformations between 126% and 138%, and tensile stresses between 2.45 MPa and 3.13 MPa, values significantly lower than those of natural rubber, which can reach elongations above 500%. However, a 46% improvement in maximum elongation was observed compared to previous studies on recycled rubber. This is attributed to the fact that the moisture-activated polyurethane binder allows for greater adhesion between the matrix particles, resulting in increased flexibility compared to the recycled rubber matrices used by other authors. Regarding the M8, M9, and M10 matrices with additives, it was found that calcium lignosulfonate reduced particle adhesion, while large amounts of cellulose fiber excessively increased stiffness. Consequently, these additives were excluded from the final matrix formulation.

Finally, mechanical evaluations of the seismic isolator prototypes were conducted. In the cyclic compression test, it was observed that the vertical stiffness of the prototypes developed in this study was higher compared to those using natural rubber and those evaluated by other authors employing recycled rubber. Specifically, the PF-PAFV15 prototype, composed of the M4 matrix reinforced with fiberglass, exhibited 30% greater stiffness than the natural rubber prototype and 61% greater stiffness than the recycled rubber prototypes reported in other studies. These results indicate that the evaluated matrix offers a stiffer response to vertical loads. Furthermore, the monotonic compression test concluded that while the M4 matrix alone presents lower vertical stiffness compared to natural rubber, when combined with fiberglass as a reinforcement in the prototypes, it achieves significantly greater stiffness. This suggests that the combination of the M4 matrix with fiberglass not only improves the performance of the seismic isolators but may even surpass the performance of natural rubber prototypes.

Based on the results from the cyclic shear test, it can be concluded that the PF-PAFV15 prototype emerged as the most efficient and robust device among those evaluated in this study, completing all deformation cycles without failure, unlike the other prototypes, which experienced significant failures at 50% deformation, including the recycled rubber prototype evaluated by other authors. Compared to the natural rubber prototype, the PF-PAFV15 achieved a damping modulus 44% higher at 100% deformation, indicating a greater capacity to dissipate energy under extreme cyclic loading conditions. This increase in damping is necessary for enhancing the efficiency of seismic isolation, as it reduces the transfer of seismic energy to the structure, thereby providing greater protection during severe seismic events.

In conclusion, this research validated the M4 matrix through mechanical testing in seismic isolator prototypes, demonstrating its ability to provide superior vertical stiffness and damping modulus compared to other materials, including those based on natural rubber. These results suggest that the M4 matrix, particularly when combined with reinforcements such as fiberglass, has significant potential to enhance the performance of seismic isolators. However, to confirm its effectiveness and applicability in real-world conditions, additional testing on full-scale seismic isolators is necessary. This will allow for the evaluation of its behavior under more representative seismic field loads and conditions. Additionally, it will enable the estimation of costs, and consequently, the economic impact of its manufacturing process.

## Figures and Tables

**Table 1:** Abbreviations.

Abbreviation	Term
SREI	Steel-reinforced elastomeric isolators
FREI	fiber-reinforced elastomeric isolators
U-FREI	Unbonded Fiber Reinforced Elastomeric Isolator
RR-FREIs	Recycled Rubber-fiber-reinforced elastomeric isolators
HDR	High Damping Rubber
RC	Reclaimed-Rubber Compound
GTR	Ground Tire Rubber
U-STI	Unbonded scrap tire isolator
PB-MDI	MDI Prepolymer Binder
RR	Recycled Rubber
BP	Bidirectional polyester

**Table 2:** Particle size distribution GTR.

Sieve	Diameter (mm)	Classification
#50–#200	0.074–0.297	Fine
#10–#50	0.595–2.00	Medium
#10	>2.00	large

**Table 3:** Properties of reinforcement fibers.

Material	ID	Thickness [mm]	Elasticity Module [MPa]
Bidirectional polyester [24]	BP	1.1	1165
Glass fiber mesh with epoxy resin [25]	FVE	1.36	2280

**Table 4:** Adhesion results following the ISO36 and EN1465 [22].

Adhesive	ID	Average Adhesion Strength ISO36 (N/mm)	Average Shear Adhesion Strength EN1945 (N/mm^2^)
MDI prepolymer binder	AA (PB-MDI)	1768	2527
Hybrid mounting adhesive [22]	PF	0.541	0.790

**Table 5:** Matrices evaluated.

Specimen	Particle Size Distribution		PB-MDI (%)	Temperature (°C)	Calcium Lignosulfonate (%)	Cellulose Fibers (%)
	Fine (%)	Medium (%)				
M1	100%	0%	15	140	0	0
M2	75%	25%	15	140	0	0
M3	50%	50%	15	140	0	0
M4	25%	75%	15	140	0	0
M5	0%	100%	15	140	0	0
M6	25%	75%	20	140	0	0
M7	25%	75%	10	140	0	0
M8	25%	75%	15	140	3	0
M9	25%	75%	15	140	0	3
M10	25%	75%	15	140	0	5

**Table 6:** Specimens Description.

Prototype	Adhesive	Mix	Reinforcement
Ref [2]	-	Natural	BP [24]
AA-PAP10	AA	M7	BP [24]
AA-PAP15	AA	M4	BP [24]
AA-PAFV15	AA	M4	FVE [25]
PF-PAFV10	PF [25]	M7	FVE [25]
PF-PAFV15	PF [25]	M4	FVE [25]

**Table 7:** Hardness results for the Matrices.

Specimen	1	2	3	Average	Standard Deviation (SD)
Natural [6]	-	-	-	57	-
Ref [21]	-	-	-	74.9	-
M1	61.4	60.2	60.2	60.6	0.6
M2	62	63.2	62	62.4	0.6
M3	64.4	66	65	65.13	0.7
M4	64.6	65.2	65.8	65.2	0.5
M5	66.2	65.8	65.4	65.8	0.3
M6	64.6	67.4	67.2	66.4	1.3
M7	60.6	62	63.8	62.13	1.3
M8	63	61.4	61.2	61.87	0.8
M9	67	68.2	68.2	67.8	0.6
M10	68.6	70.4	69.8	69.6	0.7

**Table 8:** Shear results for the matrices.

Specimen	γs = 20%		γs = 50%	
	G (MPa)	Β (%)	G (MPa)	Β (%)
Natural [6]	1.20	7.50	0.78	6.00
Ref [22]	1.70	3.80	1.00	4.5
M1	2.31	3.31	1.88	2.86
M2	2.30	3.37	1.87	2.65
M3	2.46	3.48	1.88	3.13
M4	2.28	3.75	1.84	2.96
M5	2.57	3.41	2.03	2.82
M6	2.85	3.84	2.18	3.39
M7	2.11	3.33	1.68	2.95
M8	2.01	4.43	1.31	4.13
M9	2.71	3.62	1.98	3.78
M10	3.20	2.93	2.25	3.06

**Table 9:** Residual compression test results.

Specimen	1	2	3	4	5	Average
t_0_ (mm)	6.21	6.31	6.20	6.50	6.71	6.39
t_i_ (mm)	5.76	5.86	5.77	5.95	6.10	5.89
tn (mm)	4.7	4.7	4.7	4.7	4.7	4.70
Def (%)	29.8	28.0	28.7	30.6	30.3	29.5

**Table 10:** Tension test results. Maximun stress and strain.

Specimen	Maximum Stress [MPa]	Maximum Strain [%]
1	2.5	136.0
2	3,1	138.0
3	3.0	133.0
4	2.5	126.0
Average	2.8	133.0
Standard Deviation (SD)	0.3	4.5
Ref [21]	2.7	74
Natural matrix [6]	17	630

**Table 11:** Cyclic compression results of the different prototypes.

Prototype	Kvdexp [KN/mm]	Kvdth [KN/mm]	Difference [%]	Hi [mm]	Hf [mm]	εres [%]
Ref Natural [2]	19.5	20	2.50%	-	-	-
Ref recycled [14]	10.8	20	46.00%	-	-	-
AA-PAP10	27.18	33	17.65%	45.25	44.98	0.60%
AA-PAP15	28.65	35	18.15%	45.65	45.2	0.99%
AA-PAFV15	50.44	53	4.83%	45.23	45.12	0.24%
PF-PAFV10	43.78	49	10.65%	45.23	45.09	0.31%
PF-PAFV15	45.11	53	14.89%	46.24	46.04	0.43%

**Table 12:** Cyclic shear test results. FD: fault deformation.

	Kh (N/mm) for Different Deformation					Damping (%) for Different Deformation				
Prototype	25%	50%	67%	100%	75%	25%	50%	67%	100%	75%
Ref Natural [2]	190.00	124.00	100.00	77.00	81.00	19.21%	15.21%	12.82%	12.63%	13.20%
Ref recycled [21]	537.29	365.03	FD	-	-	12.62%	17.02%	FD	-	-
AA-PAP10	440.00	300.00	FD	-	-	14.85%	20.52%	FD	-	-
AA-PAP15	455.00	308.00	FD	-	-	15.50%	19.71%	FD	-	-
AA-PAFV15	646.02	FD	-	-	-	13.46%	FD	-	-	-
PF-PAFV10	344.45	255.21	212.09%	FD	-	12.31%	13.20%	15.02%	FD	-
PF-PAFV15	429.38	308.68	358.19%	188.59	166.26	12.12%	13.50%	14.46%	22.70%	22.68%

## Data Availability

Data will be made available on request.
